# Comparative Analysis of Amino Acids, Nucleosides, and Nucleobases in *Thais clavigera* from Different Distribution Regions by Using Hydrophilic Interaction Ultra-Performance Liquid Chromatography Coupled with Triple Quadrupole Tandem Mass Spectrometry

**DOI:** 10.1155/2015/394526

**Published:** 2015-07-28

**Authors:** Yahui Ge, Yuping Tang, Sheng Guo, Xin Liu, Zhenhua Zhu, Pei Liu, Jin-ao Duan

**Affiliations:** Jiangsu Collaborative Innovation Center of Chinese Medicinal Resources Industrialization, and National and Local Collaborative Engineering Center of Chinese Medicinal Resources Industrialization and Formulae Innovative Medicine, Nanjing University of Chinese Medicine, Nanjing 210023, China

## Abstract

*Thais clavigera*, as function food, is distributed widely along the coasts of China. To compare and tap its potentially nutritional and functional values, hydrophilic interaction ultra-performance liquid chromatography coupled with triplequadrupole tandem mass spectrometry (HILIC-UPLC-TQ-MS/MS) was used for simultaneous identification and quantification of amino acids, nucleosides, and nucleobases in the extracts of *T. clavigera* from 19 sea areas in China, and a PCA was further performed for comparing their content variation in different distribution regions. The total contents of amino acids varied from 116.74 mg/g to 298.58 mg/g being higher than contents of nucleosides and nucleobases that varied from 2.65 mg/g and 20.49 mg/g. Among the habitats, Hainan province had content advantages on others. By PCA, samples collected from different regions were classified into three groups. For specific constituents, lysine accounted for large part of essential amino acids; glycine and taurine also play important roles in the delicate taste and health care function of it. Inosine takes up most of total contents of nucleosides and nucleobases. These results provided good data for establishing quality standard of *T. clavigera* related products and their further development and utilization.

## 1. Introduction

*Thais clavigera*, a taupe shuttle-like conch, inhabits in the mesolittoral zone, on the rocks, and under the pebbles along the coasts from south to north in China and most coasts in Japan [1]. In recent years, it is usually applied as indicator of heavy metal pollution especially organotin in ocean [2, 3]. It has also been used as Chinese medicine for over 1000 years, and all of its parts including the shell, flesh, and operculum of* T. clavigera* could be used as medicine, which are called liaoluo, hailuo, and hailuoyan, respectively [4]. Since the flesh of* T. clavigera* tastes delicious, it has been often used as dishes by locals since ancient time.

As there has been a certain amount of researches on nutrient and functional composition of some economic snails in ocean [5], the studies on these components in* Thais* including* T. clavigera* are quite few. In previous studies, nutritional composition of wild* T. clavigera* in only a region (intertidal zone of Dongji Island in Zhoushan, China) was analyzed by visible spectrophotometry. The results showed that it contains rich proteins, fatty acids, sterols, and mineral elements. And 17 amino acids were checked out in the species, which accounted for 60.17 g of the total dry weight; essential amino acids accounted for 35.57% of the total amino acids [6], which indicates that* T. clavigera* is high-quality protein source. As we all know, amino acids are basic structure units of biomacromolecules such as protein and enzyme. Moreover, the free amino acids associated with* Buthus martensii*,* Cervus nippon *Temminck, and* Calculus bovis* [7–9] have been paid considerable attention [10–13]. As for the nucleosides and nucleobases, since more and more researches in* Ganoderma lucidum* [14],* Cordyceps sinensis* [15], and* Isatis indigotica* [16] have been carried out, they have also been proven to be important nutritional and functional compositions [17–19]. For a nutrient and functional perspective, it is essential to determine the amino acids, nucleosides, and nucleobases in function foods for the quality control and potential useful value. However, the amino acids were usually determined by visible spectrophotometry after derivatization with ninhydrin in previous study [6], which has low sensitivity and complex pretreatment procedure. In the present study, we collected* T. clavigera *samples from 19 areas along the coasts in China. To determine these hydrophilic compounds in two categories, hydrophilic interaction ultra-performance liquid chromatography coupled with triple quadrupole tandem mass spectrometry (HILIC-UPLC-TQ-MS/MS) was used for simultaneous identification and quantification of amino acids, nucleosides, and nucleobases in the extracts of* T. clavigera*. After content analysis, a PCA method was further performed for comparing their content variation in different distribution regions.

## 2. Experiment

### 2.1. Chemicals and Reagents

Ammonium formate (Shanghai Lingfeng Chemical Reagent Co., Ltd., Shanghai, China), ammonium acetate (Sinopharm Chemical Reagent Co., Ltd., Shanghai, China), formic acid (Merck Millipore, Germany), and acetonitrile (Tedia Company Inc., United States) were of analytical grade. The deionized water (H_2_O) was purified by a superpurification system (EPED Technology Development, Nanjing, China). 41 standards including adenosine-5′-monophosphate (**1**), inosine (**2**), guanosine (**3**), thymidine (**4**), 2′-deoxyuridine (**5**), 2′-deoxyinosine (**6**), cytidine-5′-monophosphate (**7**), 2′-deoxyadenosine-5′-monophosphate (**8**), 2′-deoxycytidine (**9**), 2′-deoxyguanosine (**10**), thymine (**11**), adenine (**12**), cytidine (**13**), uracil (**14**), guanine (**15**), glycine (**17**), GABA (**18**), asparagine (**27**), glutamine (**28**), citrulline (**30**), hydroxyproline (**32**), taurine (**35**), and ornithine (**37**) were purchased from Sigma (St. Louis, MO, USA). Standards including xanthine (**16**), leucine (**19**), isoleucine (**20**), phenylalanine (**22**), tryptophan (**23**), alanine (**24**), threonine (**25**), serine (**26**), glutamate (**29**), proline (**31**), tyrosine (**34**), valine (**36**), aspartate (**38**), lysine (**39**), histidine (**40**), and arginine (**41**) were obtained from the National Institute for the Control of Pharmaceutical and Biological Products, Beijing, China. A chemical standard of cysteine (**33**) was obtained from Aladdin Chemical, Shanghai, China. A reference of methionine (**21**) was purchased from Sinopharm Chemical Reagent, Beijing, China. The purity of each compound was >98%, determined by UPLC analysis.

### 2.2. Sample Materials

The samples of* T. clavigera* are collected from 19 different habitats of China in August 2014. Details of each sample are listed in Table 1. The* T. clavigera* were collected from the habitats and identified by Professor Ding Shaoxiong (Xiamen University, Fujian Province, China). After collection, the animals were kept in −80°C refrigerator. The voucher specimens were deposited in Nanjing University of Chinese Medicine, China.

### 2.3. Preparation of Standard Solutions

A mixed standard stock solution containing the reference compounds 1–41 dried to constant weight was prepared in methanol/water (9 : 1, v/v). Working standard solutions for calibration curves were prepared by diluting the mixed standard stock solution with 10% methanol at different concentrations.

### 2.4. Sample Preparation

After unfreezing at the temperature of 4°C and removing the shell, the flesh of* T. clavigera* was freeze-dried by the vacuum freeze dry systems (Labconco, United States), and then they were weighed and smashed, respectively. One gram of each dry sample was accurately weighed into 50 mL conical flask, and 40 mL distilled water was added. All of the mixtures were precisely weighed and placed into an ultrasonic bath (40 kHz) for 60 min at room temperature and distilled water was added to compensate for the loss of water during extraction. The supernatants of extracting solution were separated after centrifugation (13000 r/min) for 10 minutes and then eliminated protein by adding acetonitrile to double volume and then stored at 4°C and filtered through 0.22 *μ*m cellulose membrane filters prior to injection.

### 2.5. UPLC-TQ-MS/MS Condition

A Waters ACQUITY UPLC system (Waters, Milford, MA, USA) equipped with a quaternary pump solvent management system, an online degasser, and an autosampler was used for analysis. The chromatographic separation was carried out on an ACQUITY UPLC BEH amide column (2.1 mm × 100 mm, 1.7 *μ*m). The raw data were acquired and processed with MassLynx 4.1 software. The mobile phase was composed of A (5 mmol/L ammonium formate, 5 mmol/L ammonium acetate, and 0.2% formic acid in aqueous solution) and B (1 mmol/L ammonium formate, 1 mmol/L ammonium acetate, and 0.2% formic acid in acetonitrile) with a gradient elution: 0–3 min, 10% A; 3–9 min, 10–18% A; 9–15 min, 18–20% A; 15-16 min, 20–46% A; 16–18 min, 46% A. The flow rate and column temperature were set at 0.4 mL/min 35°C.

The mass spectrometry analysis was performed on Xevo Triple Quadrupole MS (Waters Corp., Milford, MA, USA) equipped with an electrospray ionization (ESI) source operating in positive ionization mode. And the conditions of the ESI source were set as follows: the desolvation gas flow rate was set to 1000 L/h, the desolvation temperature was set at a temperature of 350°C, the cone gas flow rate was set at 20 L/h, the source temperature was set at 120°C, and the cone voltage and collision energy were set depending upon the MRM for each compound. Data were collected in MRM mode by screening parent and daughter ions simultaneously.

## 3. Results and Discussion

### 3.1. Optimization of the Sample Preparation

In this study, in order to obtain the best extraction efficiency, variable factors during the extraction including solvent (water, aqueous methanol of different concentrations), solvent volume (10, 20, 30, 40, 50, and 60 mL), extraction temperature (20, 40, 60, 80, and 100°C), extraction methods (refluxing and ultrasonication), and extraction time (10, 20, 30, 40, 50, and 60 min) were optimized. During the optimization, every single parameter was evaluated on condition that others were set at default. The results showed that the ultrasonic bath was better than reflux extraction. And according to the peak area as evaluated criteria, it revealed that ultrasonic bath with the solvent of 50 mL water at room temperature for 60 min was the best condition.

### 3.2. Optimization of UPLC-TQ-MS/MS Conditions

**Figure 1:**
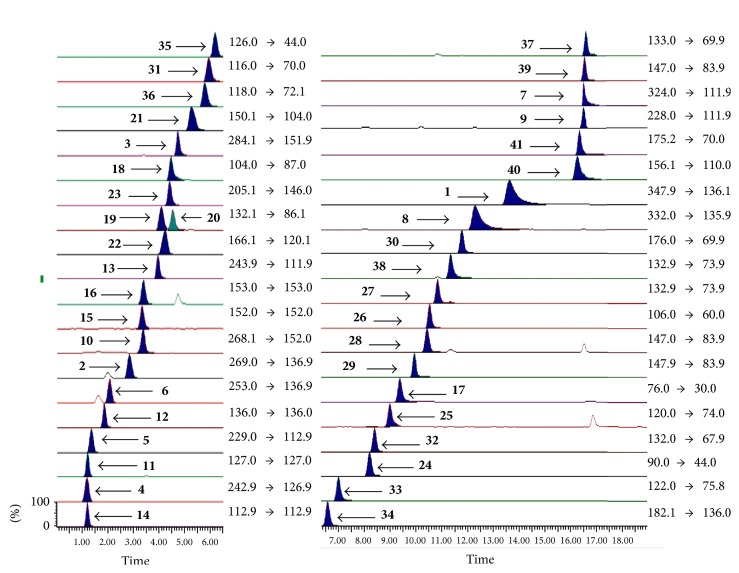
UPLC-TQ-MS/MS multiple-reaction monitoring (MRM) chromatograms of the samples. The compound numbers on the chromatograms were adenosine-5′-monophosphate (**1**), inosine (**2**), guanosine (**3**), thymidine (**4**), 2′-deoxyuridine (**5**), 2′-deoxyinosine (**6**), cytidine-5′-monophosphate (**7**), 2′-deoxyadenosine-5′-monophosphate (**8**), 2′-deoxycytidine (**9**), 2′-deoxyguanosine (**10**), thymine (**11**), adenine (**12**), cytidine (**13**), uracil (**14**), guanine (**15**), xanthine (**16**), glycine (**17**), GABA (**18**), leucine (**19**), isoleucine (**20**), methionine (**21**), phenylalanine (**22**), tryptophan (**23**), alanine (**24**), threonine (**25**), serine (**26**), asparagine (**27**), glutamine (**28**), glutamate (**29**), citrulline (**30**), proline (**31**), hydroxyproline (**32**), cysteine (**33**), tyrosine (**34**), taurine (**35**), valine (**36**), ornithine (**37**), aspartate (**38**), lysine (**39**), histidine (**40**), and arginine (**41**), respectively.

To achieve better results of analysis, the conditions of UPLC were optimized. The separation was investigated on an ACQUITY BEH Amide (100 mm × 2.1 mm, 1.7 *μ*m) after comparison of two columns, an ACQUITY BEH C18 (100 mm × 2.1 mm, 1.7 *μ*m), and an ACQUITY BEH Amide (100 mm × 2.1 mm, 1.7 *μ*m). Acetonitrile with better elution ability, separation selectivity, and peak shape compared to methanol was used as organic phase. In addition, the dissolution of ammonium acetate and ammonium formate in acetonitrile could improve the separation of amino acids, nucleosides, and nucleobases for HPLC analysis. Meanwhile, formic acid was also used to inhibit solute ionization to improve the shape of peak. Consequently, acetonitrile mixed with 1 mmol/L ammonium formate, acetate, and 0.2% formic acid were chosen for organic phase, and deionized water mixed with 5 mmol/L ammonium formate, ammonium acetate, and 0.2% formic acid were chosen for aqueous phase. The flow rate and column temperature were both optimized, and they were set at 0.4 mL/min and 35°C, respectively. The typical chromatograms of the 40 analytes are presented in Figure 1.

As for MS/MS condition, all of the compounds were examined separately in direct infusion mode by full-scan MS method in both positive and negative ionization modes for better analysis. It was found that both higher sensitivity and clearer mass spectra were obtained in the positive ion mode compared to the negative ion mode.

### 3.3. Method Validation

The proposed UPLC method was validated by determining the linearity, LOD, LOQ, precision, repeatability, stability, and recovery. And all the calibration curves exhibited good linear regressions (*r* ^2^ > 0.9919) within the test ranges. The LOD was determined at signal-to-noise (S/N) ratio of 3, and the LOQ was determined at S/N ratio of 10, each of which was 0.003–0.112 *μ*g/mL and 0.008–0.352 *μ*g/mL, respectively. The overall RSDs of intraday precisions and interday precisions were <3.48% and <3.24%, respectively. The RSDs of repeatability and stability assessed were <3.42% and <4.89%, respectively. The recoveries of the 41 compounds were in the range of 92.62%–110.51% and the RSDs were <4.10%. Moreover, it was proved to have no significant matrix effects in relatively complex functional food matrices within 24 h. As a result, the established method for simultaneous determination of 41 amino acids, nucleosides, and nucleobases in* T. clavigera* was accurate, sensitive, and repeatable.

### 3.4. Characteristics of the Amino Acids, Nucleosides, and Nucleobases in Samples

**Figure 2:**
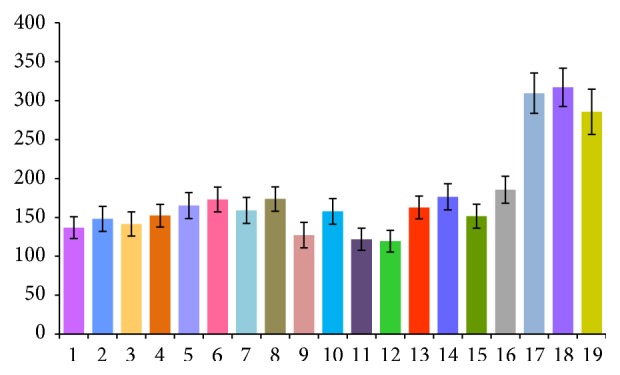
The total content of 41 compounds in different samples. Samples 1–19 are collected from Yingkou, Liaoning; Dalian (Dachangshan Island), Liaoning; Dalian (Clam Island), Liaoning; Yantai, Shandong; Weihai, Shandong; Qingdao, Shandong; Lianyungang (Lian Island), Jiangsu; Lianyungan (Qinshan Island), Jiangsu; Wenzhou (Nanfu Island), Zhejiang; Wenzhou, Zhejiang; Ningbo (Meishan Island), Zhejiang; Ningbo (Yushan Island), Zhejiang; Fuzhou (Langqi Island), Fujian; Fuzhou (Dongjia Island), Fujian; Xiamen (Dadeng Island), Fujian; Xiamen (Gulangyu Island), Fujian; Sanya (Sanya Bay), Hainan; Sanya (Wuzhizhou Island), Hainan and Haikou, Hainan.

The amino acids, nucleosides, and nucleobases in* T. clavigera* collected from 19 different habitats in China were analyzed. The contents of the 41 compounds are listed in Table 2, which indicate that there is a large difference among different samples. The results showed that the samples are rich in amino acids, nucleosides, and nucleobases and the total contents vary from 119.41 mg/g (sample 12) to 317.10 mg/g (sample 18). The total contents of 41 compounds in different samples are showed in Figure 2. The results showed that* T. clavigera* collected from Hainan was mostly higher in nutritional value compared to the other habitats. And the shape and appearance of the* T. clavigera* of Hainan were different, which were much bigger and purer. All of these may be attributed to many factors such as much better marine environments surrounding Hainan province.

The 19 samples tested by the methods mentioned above mainly contain all the 16 nucleosides and nucleobases determined in the study. However, comparing to the amino acids, nucleosides and nucleobases were mostly of microgram magnitude, and total contents of them in different samples vary from 2.65 mg/g to 20.49 mg/g. The contents of* T. clavigera* collected from different sea areas were in the following order: Hainan > Shandong > Liaoning > Jiangsu > Fujian > Zhejiang. However, the quantities of all nucleosides and nucleobases we have determined in the test in one sample have no obvious differences. The samples collected from Hainan showed great advantages, which resulted in the total contents of samples from Hainan being almost ten times as much as the lowest one from other habitats. As for the specific constituents, the contents of inosine varied from 0.31 mg/g to 3.61 mg/g taking up 7.43% to 38.21% of total contents of nucleosides and nucleobases in the samples.

As for the amino acids, the quantities of detected amino acids except GABA and cysteine were of milligram magnitude per gram of the samples. The total contents of amino acids in the samples were between 116.74 mg/g and 298.58 mg/g, and the contents of* T. clavigera* collected from different sea areas were sequenced as follows: Hainan > Fujian > Jiangsu > Shandong > Liaoning > Zhejiang. Among the amino acids, the contents of proteinogenic amino acids varied from 98.47 mg/g to 261.05 mg/g, and the contents of 8 essential amino acids between 38.79 mg/g and 92.72 mg/g were about 1/3 of the total contents of proteinogenic amino acids. Particularly, the content of lysine is far more than others, which are between 8.19 mg/g and 19.10 mg/g. In consideration of the good taste, flavor amino acids in* T. clavigera* were studied as well, and the results displayed that, among the 25 amino acids determined in the exam, the total contents of 22 flavor amino acids from 105.18 mg/g to 276.80 mg/g in* T. clavigera* take the most portion which are over 90% of the total contents. Due to the high contents of essential amino acids and flavor amino acids,* T. clavigera* is high in nutritious value with good taste. Besides the proteinogenic amino acids, five nonprotein amino acids were also found in* T. clavigera*. The total content of nonprotein amino acids in* T. clavigera* has the highest amount of taurine, which varied from 8.05 mg/g to 20.78 mg/g in the samples. And ornithine as nonprotein amino acid second only to taurine varied from 3.44 mg/g to 11.91 mg/g.

### 3.5. Classification of Samples Using PCA Based on the Contents

**Figure 3:**
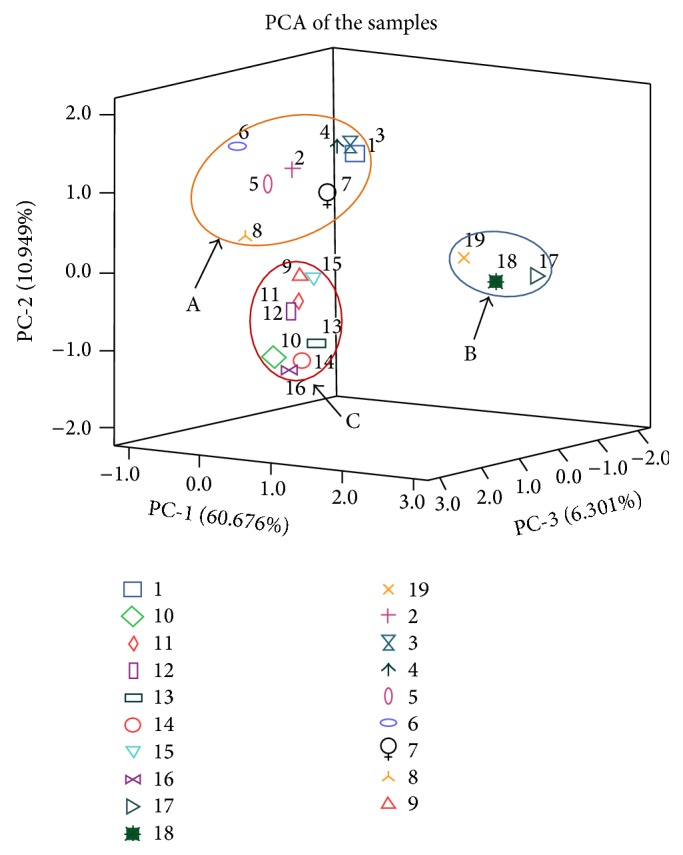
The PCA of the* Thais clavigera* Samples 1–19 are collected from Yingkou, Liaoning; Dalian (Dachangshan Island), Liaoning; Dalian (Clam Island), Liaoning; Yantai, Shandong; Weihai, Shandong; Qingdao, Shandong; Lianyungang (Lian Island), Jiangsu; Lianyungan (Qinshan Island), Jiangsu; Wenzhou (Nanfu Island), Zhejiang; Wenzhou, Zhejiang; Ningbo (Meishan Island), Zhejiang; Ningbo (Yushan Island), Zhejiang; Fuzhou (Langqi Island), Fujian; Fuzhou (Dongjia Island), Fujian; Xiamen (Dadeng Island), Fujian; Xiamen (Gulangyu Island), Fujian; Sanya (Sanya Bay), Hainan; Sanya (Wuzhizhou Island), Hainan and Haikou, Hainan.

**Figure 4:**
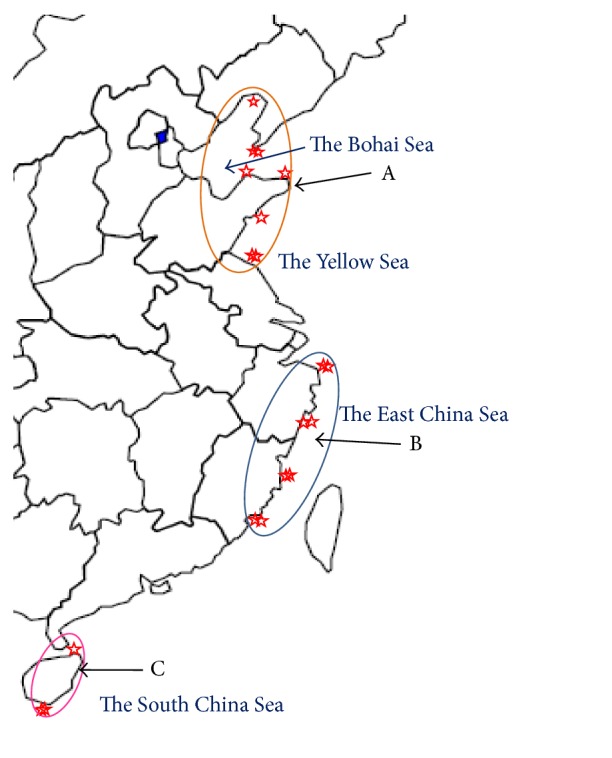
The regional distribution of* Thais clavigera*.

To explore the 25 amino acids and 16 nucleosides and nucleobases in the samples from different habitats, principal component analysis (PCA) was performed on the basis of the contents of the 41 compounds. According to the results of PCA analysis, the first three principal components (PC1, PC2, and PC3) with the total proportion of 77.93% were extracted for further information mining. And all components of the three accounted for 60.68% (PC1), 10.95% (PC2), and 6.30% (PC3). The components loading matrix is shown in Table 3. According to the loadings, PC1 had good correlations with the most compounds especially those such as adenosine-5′-monophosphate, adenine, xanthine, GABA, threonine, serine, hydroxyproline, and histidine. And PC2 had good correlations with analytes of thymidine, 2′-deoxycytidine, guanine, and lysine whereas PC3 had good correlations with analytes of cytidine and aspartate. The sample scatter plot is shown in Figure 3 where each sample is represented as a marker. It was noticeable that the samples were clearly clustered into three clusters including cluster A (the Bohai Sea and the Yellow Sea including samples 1, 2, 3, 4, 5, 6, 7, and 8 collected from Liaoning, Shandong, and Jiangsu), cluster B (the East China Sea including samples 9, 10, 11, 12, 13, 14, and 15 collected from Zhejiang and Fujian), and cluster C (the South China Sea including samples 17, 18, and 19 collected from Hainan). The regional distribution of* T. clavigera* is shown in Figure 4. From the plot, it turned out that there are significant differences between the* T. clavigera* samples collected from different geographical environment.

### 3.6. Discussion

The determination results of amino acids showed that the contents of lysine are between 8.19 mg/g and 19.10 mg/g accounting for 5.22–12.11% in our study, which was more than that in Dongji Island (4.91%) by previous report [6]. As we all know, in essential amino acids, lysine is relatively less in grain. As a consequence, the high contents of lysine in* T. clavigera* mean that people who eat grain as staple food or who eat it often can increase the intake of lysine and balance the essential amino acids in the body [20, 21]. We have recognized that the microbial catabolism of amino acids produces flavor compounds of importance for foods [22]. Aspartate, glutamate, glycine, and alanine are present as amino acids with characteristic of umami. Among the 22 flavor amino acids determined in* T. clavigera*, their total contents were varied from 25.21% to 37.99%, which could fully explain the delicate flavour of* T. clavigera*. As functional food, it has to be mentioned that there are five nonprotein amino acids, which account for 7.97–17.26%. Taurine relating to various biological processes including development of the central nervous system, membrane stabilization, and immunity appeared to be abundant in* T. clavigera* [23–25]. Ornithine second to taurine also plays an important role in metabolism. It is the intermediate product of ureogenesis as well as precursor substance of amino acids such as citrulline and histidine metabolism. It has come to public attention in recent years due to its multifunctional health care function especially the protection of liver [26, 27].

Although there have researches about determination of amino acids in* T. clavigera*, there are few methods on nucleosides and nucleobases. However, according to the results, the nucleosides and nucleobases in* T. clavigera* are various, and the contents are balanced. Among the contents, inosine, which is widely distributed in animals, takes up the most of the total nucleosides and nucleobases contents. Meanwhile, inosine is reported to protect liver [28] and inosinic acid is studied to be fresh aid related to the delicious taste [29].

In this study, it is the first time that HILIC-UPLC-TQ-MS/MS has been utilized for the simultaneous determination of these bioactive compounds in* T. clavigera* from 19 habitats as a sea snail. The results showed that, as traditional seafood,* T. clavigera* has excellent source of amino acids, nucleosides, and nucleobases with great nutritional and functional values. Importantly, inosine, lysine, glycine, and taurine in* T. clavigera* as top contents in each categories could be recognized as makers for establishing quality standards. And these research results also provided good data for establishing quality standard of* T. clavigera* even* Thais* for the further development and utilization of the marine organism.

## Figures and Tables

**Table 1:** Samples of *Thais clavigera*.

Sample	Habitat (China)
1	Yingkou, Liaoning
2	Dachangshan Island, Dalian, Liaoning
3	Clam Island, Dalian, Liaoning
4	Yantai, Shandong
5	Weihai, Shandong
6	Qingdao, Shandong
7	Lian Island, Lianyungang, Jiangsu
8	Qinshan Island, Lianyungang, Jiangsu
9	Nanfu Island, Wenzhou, Zhejiang
10	Wenzhou, Zhejiang
11	Meishan Island, Ningbo, Zhejiang
12	Yushan Island, Ningbo, Zhejiang
13	Langqi Island, Fuzhou, Fujian
14	Dongjia Island, Fuzhou, Fujian
15	Dadeng Island, Xiamen, Fujian
16	Gulangyu Island, Xiamen, Fujian
17	Sanya Bay, Sanya, Hainan
18	Wuzhizhou Island, Sanya, Hainan
19	Haikou, Hainan

Each sample was collected as three batches from the origins.

**Table 2:** Contents (mg/g) of amino acids, nucleosides, and nucleobases in *Thais clavigera*.

Analytes	Sample${}^{\text{a}}\overset{A}{\underset{A}{\sum }}$ (mg/g, *n* = 3)									
	1	2	3	4	5	6	7	8	9	10

Adenosine-5′5′^*A*^-monophosphate	0.17 ± 0.02	0.18 ± 0.02	0.20 ± 0.02	0.18 ± 0.03	0.18 ± 0.03	0.21 ± 0.05	0.16 ± 0.02	0.15 ± 0.03	0.15 ± 0.00	0.15 ± 0.03
Inosine	1.80 ± 0.29	0.44 ± 0.05	1.55 ± 0.06	1.92 ± 0.06	1.00 ± 0.19	0.41 ± 0.03	2.54 ± 0.31	1.30 ± 0.16	0.93 ± 0.14	0.31 ± 0.03
Guanosine	0.09 ± 0.01	0.09 ± 0.01	0.17 ± 0.02	0.08 ± 0.02	0.33 ± 0.07	0.13 ± 0.07	0.10 ± 0.02	0.33 ± 0.04	0.15 ± 0.02	0.05 ± 0.02
Thymidine	0.85 ± 0.02	0.98 ± 0.10	0.92 ± 0.09	0.73 ± 0.06	0.63 ± 0.13	1.11 ± 0.31	0.18 ± 0.02	2.00 ± 0.21	+^b^	0.15 ± 0.02
2′-Deoxyuridine	0.22 ± 0.02	0.53 ± 0.04	0.15 ± 0.02	0.16 ± 0.02	0.18 ± 0.01	0.45 ± 0.05	+	0.15 ± 0.03	0.16 ± 0.03	0.70 ± 0.17
2′-Deoxyinosine	0.53 ± 0.06	0.16 ± 0.02	0.64 ± 0.08	0.41 ± 0.05	0.44 ± 0.06	0.22 ± 0.04	0.40 ± 0.08	0.46 ± 0.06	0.35 ± 0.05	0.61 ± 0.07
Cytidine-5′-monophosphate	0.19 ± 0.05	0.16 ± 0.01	0.20 ± 0.04	0.16 ± 0.02	0.17 ± 0.03	0.19 ± 0.04	0.17 ± 0.04	0.17 ± 0.02	0.16 ± 0.01	0.18 ± 0.02
2′-Deoxyadenosine-5′-monophosphate	0.16 ± 0.02	0.27 ± 0.02	0.17 ± 0.02	0.24 ± 0.09	0.17 ± 0.04	0.26 ± 0.03	0.12 ± 0.01	0.17 ± 0.02	0.12 ± 0.00	0.15 ± 0.04
2′-Deoxycytidine	0.36 ± 0.05	0.17 ± 0.02	0.56 ± 0.06	0.43 ± 0.02	0.43 ± 0.03	0.28 ± 0.05	0.39 ± 0.06	0.44 ± 0.00	0.28 ± 0.01	0.15 ± 0.00
2′-Deoxyguanosine	1.04 ± 0.27	0.58 ± 0.16	1.81 ± 0.09	0.66 ± 0.16	0.76 ± 0.05	0.77 ± 0.03	0.59 ± 0.22	0.81 ± 0.02	0.12 ± 0.12	0.21 ± 0.14
Thymine	0.73 ± 0.05	0.36 ± 0.09	0.72 ± 0.06	1.88 ± 0.21	1.75 ± 0.20	0.40 ± 0.03	0.85 ± 0.08	0.82 ± 0.07	+	0.30 ± 0.05
Adenine	0.26 ± 0.03	0.28 ± 0.07	0.18 ± 0.02	0.14 ± 0.07	0.12 ± 0.01	0.24 ± 0.01	0.22 ± 0.11	0.22 ± 0.04	+	0.19 ± 0.03
Cytidine	0.07 ± 0.03	0.09 ± 0.01	0.08 ± 0.01	0.07 ± 0.02	0.55 ± 0.05	0.85 ± 0.04	+	0.52 ± 0.00	+	+
Uracil	+	0.74 ± 0.06	+	0.35 ± 0.04	+	0.81 ± 0.03	0.51 ± 0.05	0.45 ± 0.02	+	0.63 ± 0.03
Guanine	0.11 ± 0.02	0.95 ± 0.05	0.11 ± 0.04	0.13 ± 0.06	0.15 ± 0.01	0.96 ± 0.02	0.11 ± 0.01	0.14 ± 0.00	nd	+
Xanthine	0.20 ± 0.06	0.21 ± 0.01	0.18 ± 0.03	0.20 ± 0.04	0.23 ± 0.09	0.21 ± 0.04	0.13 ± 0.05	0.17 ± 0.00	0.14 ± 0.03	0.22 ± 0.02
Glycine	21.27 ± 2.47	13.21 ± 1.87	16.13 ± 2.42	18.07 ± 2.04	12.79 ± 1.54	12.93 ± 0.99	20.65 ± 0.78	15.25 ± 1.03	13.43 ± 1.02	25.00 ± 2.06
GABA	0.17 ± 0.02	0.15 ± 0.02	0.08 ± 0.00	0.06 ± 0.02	0.06 ± 0.01	0.09 ± 0.01	0.09 ± 0.00	0.12 ± 0.04	0.08 ± 0.01	0.06 ± 0.02
Leucine	6.11 ± 0.59	5.64 ± 1.21	7.64 ± 0.57	5.28 ± 0.31	7.78 ± 1.02	7.36 ± 1.18	5.94 ± 0.67	5.39 ± 1.02	5.03 ± 1.49	5.43 ± 0.37
Isoleucine	3.82 ± 0.34	3.72 ± 0.09	4.47 ± 1.16	2.96 ± 1.06	5.17 ± 1.08	4.52 ± 0.16	2.79 ± 0.07	5.05 ± 0.18	6.27 ± 1.27	8.54 ± 1.14
Methionine	1.37 ± 0.16	2.74 ± 0.86	1.45 ± 0.31	2.72 ± 0.55	1.04 ± 0.12	0.96 ± 0.22	2.73 ± 0.22	1.07 ± 0.26	1.70 ± 0.21	1.82 ± 0.32
Phenylalanine	4.72 ± 0.24	7.19 ± 0.53	5.49 ± 0.38	5.43 ± 1.27	8.78 ± 0.72	10.29 ± 1.03	5.78 ± 1.47	8.58 ± 0.43	5.22 ± 0.73	6.19 ± 1.15
Tryptophan	0.95 ± 0.06	1.96 ± 0.84	1.07 ± 0.24	1.10 ± 0.39	1.72 ± 0.56	2.18 ± 0.58	2.16 ± 1.32	3.24 ± 1.39	1.86 ± 0.57	3.20 ± 1.64
Alanine	11.97 ± 2.37	12.64 ± 1.81	12.92 ± 1.65	14.18 ± 0.83	18.06 ± 2.59	15.18 ± 1.42	15.46 ± 1.01	19.52 ± 0.43	12.21 ± 1.04	13.06 ± 0.27
Threonine	6.15 ± 0.27	6.73 ± 0.70	5.42 ± 0.64	5.82 ± 0.52	5.52 ± 0.83	6.34 ± 1.01	4.98 ± 0.45	5.65 ± 1.63	4.31 ± 0.43	6.16 ± 0.50
Serine	3.98 ± 0.67	4.54 ± 0.52	5.44 ± 0.29	4.03 ± 1.73	5.37 ± 0.37	6.18 ± 0.82	4.11 ± 1.09	5.33 ± 0.45	4.21 ± 0.62	5.94 ± 0.14
Asparagine	1.60 ± 0.18	1.85 ± 1.44	2.13 ± 1.04	1.76 ± 0.53	2.11 ± 0.67	2.49 ± 0.32	1.06 ± 0.11	1.10 ± 0.17	1.66 ± 0.24	1.67 ± 0.42
Glutamine	2.56 ± 0.38	3.07 ± 0.14	3.79 ± 0.23	2.32 ± 0.82	4.24 ± 0.36	4.57 ± 0.86	2.24 ± 1.08	3.58 ± 1.49	2.08 ± 1.14	2.25 ± 0.52
Glutamate	9.52 ± 0.50	15.87 ± 0.71	10.62 ± 0.38	15.54 ± 0.98	17.46 ± 1.28	19.63 ± 0.46	14.76 ± 1.05	17.67 ± 1.39	8.54 ± 0.27	9.94 ± 1.02
Citrulline	0.67 ± 0.11	0.72 ± 0.00	0.54 ± 0.01	0.62 ± 0.02	0.62 ± 0.01	0.52 ± 0.04	0.52 ± 0.02	0.56 ± 0.01	0.57 ± 0.00	0.48 ± 0.01
Proline	2.87 ± 0.12	3.14 ± 0.14	2.78 ± 0.11	3.15 ± 0.12	3.84 ± 0.09	3.28 ± 0.26	3.10 ± 0.15	4.06 ± 0.26	2.44 ± 0.19	2.88 ± 0.07
Hydroxyproline	0.50 ± 0.06	0.46 ± 0.00	0.51 ± 0.04	0.48 ± 0.02	0.48 ± 0.03	0.52 ± 0.05	0.50 ± 0.02	0.49 ± 0.01	0.24 ± 0.01	0.25 ± 0.00
Cysteine	+	+	+	+	+	+	+	+	+	+
Tyrosine	2.17 ± 0.19	3.88 ± 0.07	3.03 ± 0.08	6.92 ± 0.15	9.28 ± 0.24	5.96 ± 1.08	5.37 ± 1.80	7.29 ± 0.51	4.03 ± 1.22	4.71 ± 1.01
Taurine	14.85 ± 1.86	17.62 ± 1.34	13.25 ± 1.70	5.98 ± 0.39	8.30 ± 0.34	18.73 ± 0.24	17.36 ± 1.66	9.56 ± 1.10	10.72 ± 0.57	8.05 ± 0.39
Valine	4.65 ± 0.77	6.45 ± 0.23	5.12 ± 1.08	6.48 ± 0.51	7.86 ± 0.17	7.45 ± 1.02	6.51 ± 0.24	7.83 ± 1.14	7.66 ± 2.09	9.18 ± 1.44
Ornithine	3.91 ± 0.41	3.95 ± 0.87	3.44 ± 0.62	4.50 ± 0.19	6.13 ± 1.25	3.90 ± 1.09	4.74 ± 0.37	4.43 ± 0.76	4.35 ± 1.05	5.29 ± 0.52
Aspartate	3.85 ± 0.13	4.23 ± 0.15	5.13 ± 1.29	4.16 ± 0.78	5.48 ± 0.52	6.17 ± 1.32	6.10 ± 1.22	11.05 ± 0.56	5.79 ± 1.33	8.96 ± 1.01
Lysine	15.58 ± 1.98	14.78 ± 1.39	16.05 ± 1.14	15.72 ± 0.38	19.10 ± 1.58	17.50 ± 0.49	17.10 ± 1.28	19.80 ± 1.72	11.85 ± 0.85	13.12 ± 2.45
Histidine	3.04 ± 0.54	3.36 ± 0.82	3.79 ± 0.73	2.80 ± 0.66	3.52 ± 1.19	4.36 ± 0.44	4.14 ± 0.19	5.31 ± 0.21	2.21 ± 0.31	2.81 ± 0.56
Arginine	3.35 ± 0.23	3.75 ± 1.04	3.05 ± 1.09	4.16 ± 0.84	2.94 ± 0.62	4.13 ± 1.67	4.01 ± 0.89	3.12 ± 0.25	7.35 ± 0.87	8.56 ± 0.38
N^d^	7.06 ± 0.85	6.20 ± 0.60	7.92 ± 0.67	7.75 ± 0.83	7.33 ± 0.86	7.53 ± 0.73	6.65 ± 0.94	6.51 ± 0.58	3.21 ± 0.27	4.01 ± 0.53
A^e^	129.83 ± 13.20	141.77 ± 15.34	133.55 ± 14.75	144.39 ± 13.66	157.79 ± 15.74	165.37 ± 15.31	152.30 ± 15.71	167.12 ± 15.00	123.92 ± 16.08	153.70 ± 15.96
Total	136.90 ± 14.04	147.98 ± 15.94	141.47 ± 15.41	152.14 ± 14.49	165.12 ± 16.60	172.90 ± 16.04	158.95 ± 16.65	173.64 ± 15.57	127.13 ± 16.35	157.72 ± 16.49

Analytes	Sample (mg/g, *n* = 3)Sample (mg/g, *n* = 3)^*A*^									
	11	12	13	14	15	16	17	18	19	

Adenosine-5′5′^*A*^-monophosphate	0.21 ± 0.02	0.18 ± 0.02	0.18 ± 0.02	0.18 ± 0.01	0.16 ± 0.01	0.15 ± 0.01	0.81 ± 0.08	0.76 ± 0.07	0.82 ± 0.03	
Inosine	0.57 ± 0.06	0.75 ± 0.08	0.67 ± 0.10	0.42 ± 0.03	1.60 ± 0.21	0.84 ± 0.11	3.61 ± 0.34	2.15 ± 0.23	2.21 ± 0.19	
Guanosine	0.06 ± 0.02	0.05 ± 0.01	0.09 ± 0.02	0.06 ± 0.00	0.15 ± 0.01	0.10 ± 0.02	0.84 ± 0.01	0.40 ± 0.03	0.48 ± 0.05	
Thymidine	nd^c^	nd	+	0.18 ± 0.00	+	0.14 ± 0.01	0.57 ± 0.01	0.64 ± 0.11	0.43 ± 0.08	
2′-Deoxyuridine	+	+	0.38 ± 0.02	0.75 ± 0.02	0.19 ± 0.01	0.61 ± 0.07	0.61 ± 0.07	0.60 ± 0.12	0.61 ± 0.08	
2′-Deoxyinosine	0.21 ± 0.02	0.24 ± 0.03	0.42 ± 0.06	0.90 ± 0.09	0.69 ± 0.08	1.14 ± 0.03	2.67 ± 0.34	2.86 ± 1.01	1.92 ± 0.91	
Cytidine-5′-monophosphate	+	+	0.19 ± 0.03	0.18 ± 0.02	0.17 ± 0.01	0.18 ± 0.01	0.32 ± 0.01	0.27 ± 0.00	0.26 ± 0.02	
2′-Deoxyadenosine-5′-monophosphate	0.15 ± 0.01	0.16 ± 0.05	0.13 ± 0.02	0.16 ± 0.02	0.21 ± 0.00	0.13 ± 0.01	0.35 ± 0.03	0.37 ± 0.04	0.23 ± 0.01	
2′-Deoxycytidine	0.21 ± 0.02	0.15 ± 0.00	0.21 ± 0.03	0.16 ± 0.01	0.22 ± 0.02	0.22 ± 0.04	0.34 ± 0.00	0.41 ± 0.01	0.73 ± 0.04	
2′-Deoxyguanosine	0.13 ± 0.03	0.11 ± 0.01	0.23 ± 0.01	0.50 ± 0.01	0.12 ± 0.01	0.21 ± 0.00	3.15 ± 0.19	2.87 ± 0.22	2.25 ± 0.54	
Thymine	+	+	+	0.43 ± 0.02	+	0.35 ± 0.02	1.57 ± 0.21	1.76 ± 0.12	1.24 ± 0.42	
Adenine	+	+	0.12 ± 0.00	0.14 ± 0.01	+	0.18 ± 0.01	0.85 ± 0.03	0.67 ± 0.02	0.57 ± 0.02	
Cytidine	+	+	0.12 ± 0.02	0.09 ± 0.00	0.07 ± 0.00	+	0.08 ± 0.01	0.20 ± 0.02	0.21 ± 0.02	
Uracil	0.40 ± 0.12	0.45 ± 0.02	0.75 ± 0.03	1.24 ± 0.22	0.46 ± 0.02	1.48 ± 0.53	2.51 ± 0.66	2.81 ± 0.39	2.47 ± 0.32	
Guanine	nd	nd	+	+	+	+	0.41 ± 0.02	0.32 ± 0.02	0.27 ± 0.03	
Xanthine	0.13 ± 0.04	+	0.17 ± 0.01	0.23 ± 0.02	0.14 ± 0.01	0.22 ± 0.01	1.80 ± 0.07	1.44 ± 0.05	1.44 ± 0.05	
Glycine	11.86 ± 2.16	10.73 ± 1.06	15.31 ± 1.22	22.54 ± 3.53	11.79 ± 0.54	22.36 ± 2.02	30.29 ± 3.09	31.58 ± 2.56	30.06 ± 3.60	
GABA	0.10 ± 0.01	0.09 ± 0.00	0.20 ± 0.01	0.20 ± 0.02	0.13 ± 0.01	0.21 ± 0.01	2.47 ± 0.14	2.08 ± 0.17	1.20 ± 0.10	
Leucine	6.65 ± 0.78	5.48 ± 0.96	4.96 ± 1.86	5.70 ± 1.94	6.19 ± 1.65	6.25 ± 0.97	10.43 ± 2.34	10.58 ± 2.25	8.74 ± 1.16	
Isoleucine	5.12 ± 0.57	5.99 ± 1.22	7.00 ± 1.67	8.38 ± 1.41	3.51 ± 1.38	5.07 ± 0.70	8.78 ± 1.80	9.12 ± 0.84	8.90 ± 2.59	
Methionine	1.72 ± 0.02	1.48 ± 0.16	2.44 ± 0.57	2.64 ± 0.18	2.73 ± 0.07	2.97 ± 0.84	7.64 ± 0.65	8.44 ± 0.16	5.82 ± 1.97	
Phenylalanine	8.24 ± 1.28	7.35 ± 1.31	11.21 ± 0.54	12.94 ± 1.60	12.33 ± 1.52	16.13 ± 2.01	16.87 ± 1.49	17.44 ± 2.60	17.58 ± 1.99	
Tryptophan	1.20 ± 0.25	0.96 ± 0.16	1.14 ± 0.24	1.50 ± 0.19	1.11 ± 0.25	1.98 ± 0.49	3.31 ± 0.55	3.06 ± 0.07	3.03 ± 0.97	
Alanine	9.26 ± 0.49	8.82 ± 2.35	14.90 ± 0.26	12.03 ± 0.62	11.78 ± 0.59	13.28 ± 1.80	27.24 ± 2.16	27.08 ± 1.02	27.44 ± 2.00	
Threonine	3.85 ± 2.16	2.98 ± 0.32	4.71 ± 0.08	5.72 ± 0.14	4.55 ± 0.21	5.76 ± 0.23	17.74 ± 1.58	18.72 ± 2.06	16.63 ± 1.07	
Serine	3.50 ± 0.23	3.12 ± 0.12	4.94 ± 0.13	5.25 ± 0.32	4.22 ± 0.11	6.14 ± 0.92	13.60 ± 2.13	16.12 ± 1.14	13.30 ± 2.10	
Asparagine	0.99 ± 1.06	1.04 ± 0.85	3.12 ± 2.11	3.42 ± 0.21	1.69 ± 0.48	1.72 ± 0.21	3.72 ± 0.57	4.33 ± 0.28	2.71 ± 0.34	
Glutamine	2.07 ± 0.79	2.57 ± 1.42	4.52 ± 0.83	5.42 ± 1.51	4.99 ± 0.60	5.50 ± 0.18	8.46 ± 1.13	10.08 ± 0.72	6.49 ± 1.04	
Glutamate	8.42 ± 0.62	9.28 ± 0.16	8.91 ± 1.01	9.97 ± 0.86	8.19 ± 1.94	10.75 ± 1.65	22.86 ± 0.97	23.74 ± 2.11	21.73 ± 1.84	
Citrulline	0.35 ± 0.81	0.25 ± 0.02	1.07 ± 0.04	0.99 ± 0.05	0.94 ± 0.04	0.97 ± 0.03	2.09 ± 0.09	1.56 ± 0.04	1.56 ± 0.11	
Proline	2.68 ± 0.13	2.93 ± 0.77	5.74 ± 0.67	5.52 ± 0.95	4.73 ± 0.18	5.65 ± 1.03	6.98 ± 0.74	6.73 ± 0.21	5.48 ± 0.93	
Hydroxyproline	0.22 ± 0.01	0.21 ± 0.01	0.50 ± 0.02	0.54 ± 0.03	0.49 ± 0.02	0.52 ± 0.00	4.28 ± 0.50	4.37 ± 0.10	3.92 ± 0.33	
Cysteine	nd	nd	+	+	+	+	0.35 ± 0.00	0.35 ± 0.01	0.31 ± 0.01	
Tyrosine	5.32 ± 0.20	5.83 ± 0.05	4.27 ± 0.09	4.63 ± 0.14	9.49 ± 1.17	11.81 ± 0.96	7.36 ± 0.23	7.66 ± 0.38	7.53 ± 0.09	
Taurine	11.90 ± 0.76	11.08 ± 1.23	15.59 ± 1.18	14.89 ± 1.02	10.06 ± 0.92	7.24 ± 0.14	19.61 ± 1.35	20.78 ± 2.11	17.85 ± 1.39	
Valine	5.29 ± 0.45	6.02 ± 0.18	7.47 ± 0.79	8.59 ± 0.29	4.33 ± 0.29	5.53 ± 0.15	9.55 ± 0.51	9.64 ± 0.64	8.22 ± 1.01	
Ornithine	6.67 ± 0.50	6.64 ± 0.37	10.01 ± 0.57	3.81 ± 0.09	6.15 ± 0.84	5.62 ± 0.84	5.98 ± 0.38	5.82 ± 0.22	6.67 ± 0.76	
Aspartate	8.09 ± 0.29	7.63 ± 0.79	8.03 ± 0.44	8.88 ± 1.00	5.23 ± 1.91	9.18 ± 0.62	7.73 ± 0.33	8.53 ± 0.58	6.97 ± 0.25	
Lysine	8.19 ± 0.63	8.55 ± 0.54	12.65 ± 0.82	14.52 ± 1.25	16.40 ± 1.09	16.26 ± 1.13	15.07 ± 0.86	15.72 ± 0.81	15.45 ± 0.65	
Histidine	2.07 ± 0.61	1.85 ± 0.26	2.86 ± 0.09	2.98 ± 0.05	2.23 ± 0.30	2.85 ± 0.02	11.93 ± 0.32	12.00 ± 0.58	11.37 ± 0.29	
Arginine	5.28 ± 0.18	5.82 ± 0.37	6.98 ± 0.66	8.52 ± 0.38	15.14 ± 0.54	15.94 ± 1.07	18.76 ± 1.39	20.12 ± 2.11	20.99 ± 1.27	
N^d^	2.65 ± 0.20	2.68 ± 0.08	4.04 ± 0.23	5.70 ± 0.51	4.64 ± 0.25	6.05 ± 0.74	20.49 ± 1.94	18.52 ± 2.32	16.15 ± 2.67	
A^e^	119.11 ± 14.00	116.74 ± 13.78	158.64 ± 14.45	170.61 ± 16.33	146.74 ± 15.20	179.52 ± 16.56	289.02 ± 23.85	298.58 ± 22.32	269.38 ± 26.41	
Total	121.75 ± 14.19	119.41 ± 13.86	162.69 ± 14.68	176.31 ± 16.84	151.37 ± 15.45	185.57 ± 17.30	309.51 ± 25.79	317.10 ± 24.64	285.53 ± 29.08	

The data was presented as average of three batches of samples.

^a^Sample number.

^b^Below the limit of quantitation.

^c^Undetected.

^d^Total content of nucleosides and nucleobases.

^e^Total content of amino acids.

**Table 3:** Component loading matrix of 41 amino acids, nucleosides, and nucleobases for PCA.

Component (PC)	Analytes																				
	1	2	3	4	5	6	7	8	9	10	11	12	13	14	15	16	17	18	19	20	21
1	0.956	0.660	0.862	0.234	0.573	0.959	0.918	0.766	0.451	0.906	0.624	0.956	0.092	0.938	0.269	0.975	0.822	0.960	0.870	0.658	0.928
2	0.001	0.273	0.121	0.835	−0.360	−0.166	0.099	0.321	0.541	0.283	0.448	0.126	0.493	−0.219	0.551	−0.024	−0.172	−0.070	0.216	−0.551	−0.144
3	−0.158	−0.472	0.005	0.055	0.345	−0.112	−0.006	0.029	−0.155	−0.180	−0.082	−0.083	0.758	0.046	0.389	−0.132	−0.116	−0.157	0.068	0.115	−0.212

Component (PC)	Analytes																				
	22	23	24	25	26	27	28	29	30	31	32	33	34	35	36	37	38	39	40	41	

1	0.791	0.674	0.933	0.983	0.980	0.790	0.902	0.799	0.918	0.794	0.980	0.909	0.314	0.594	0.643	0.631	0.239	0.251	0.973	0.788	
2	−0.359	−0.052	0.150	0.053	−0.031	−0.097	−0.131	0.441	−0.160	−0.365	−0.002	0.087	−0.189	0.380	−0.181	−0.394	−0.658	0.553	0.109	−0.421	
3	0.219	0.438	0.123	−0.066	0.033	0.076	0.135	0.293	−0.097	0.134	−0.132	−0.263	0.381	−0.216	0.391	−0.144	0.469	0.457	−0.013	−0.075	
